# Merino and Merino-derived sheep breeds: a genome-wide intercontinental study

**DOI:** 10.1186/s12711-015-0139-z

**Published:** 2015-08-14

**Authors:** Elena Ciani, Emiliano Lasagna, Mariasilvia D’Andrea, Ingrid Alloggio, Fabio Marroni, Simone Ceccobelli, Juan V. Delgado Bermejo, Francesca M. Sarti, James Kijas, Johannes A. Lenstra, Fabio Pilla, the International Sheep Genomics Consortium

**Affiliations:** Dipartimento di Bioscienze, Biotecnologie, Biofarmaceutica, Università degli Studi di Bari “Aldo Moro”, Via Amendola 165/A 70126, Bari, Italy; Dipartimento di Scienze Agrarie, Alimentari e Ambientali, Università degli Studi di Perugia, Borgo XX giugno, 74 06121 Perugia, Italy; Dipartimento di Agricoltura, Ambiente e Alimenti, Università degli Studi del Molise, Campobasso, 86100 Italy; Dipartimento di Scienze Agrarie e Ambientali, Universita’ di Udine, Via delle Scienze 206, 33100 Udine, Italy; Istituto di Genomica Applicata (IGA), via J Linussio 51, 33100 Udine, Italy; Departamento de Génetica, Universidad de Córdoba, Edificio Méndel C5, Campus Rabanales, 14071 Cordoba, Spain; CSIRO Agriculture Flagship, St Lucia, Brisbane, QLD Australia; Faculty of Veterinary Medicine, Utrecht University, Yalelaan 104, 3584CM Utrecht, The Netherlands

## Abstract

**Background:**

Merino and Merino-derived sheep breeds have been widely distributed across the world, both as purebred and admixed populations. They represent an economically and historically important genetic resource which over time has been used as the basis for the development of new breeds. In order to examine the genetic influence of Merino in the context of a global collection of domestic sheep breeds, we analyzed genotype data that were obtained with the OvineSNP50 BeadChip (Illumina) for 671 individuals from 37 populations, including a subset of breeds from the Sheep HapMap dataset.

**Results:**

Based on a multi-dimensional scaling analysis, we highlighted four main clusters in this dataset, which corresponded to wild sheep, mouflon, primitive North European breeds and modern sheep (including Merino), respectively. The neighbor-network analysis further differentiated North-European and Mediterranean domestic breeds, with subclusters of Merino and Merino-derived breeds, other Spanish breeds and other Italian breeds. Model-based clustering, migration analysis and haplotype sharing indicated that genetic exchange occurred between archaic populations and also that a more recent Merino-mediated gene flow to several Merino-derived populations around the world took place. The close relationship between Spanish Merino and other Spanish breeds was consistent with an Iberian origin for the Merino breed, with possible earlier contributions from other Mediterranean stocks. The Merino populations from Australia, New Zealand and China were clearly separated from their European ancestors. We observed a genetic substructuring in the Spanish Merino population, which reflects recent herd management practices.

**Conclusions:**

Our data suggest that intensive gene flow, founder effects and geographic isolation are the main factors that determined the genetic makeup of current Merino and Merino-derived breeds. To explain how the current Merino and Merino-derived breeds were obtained, we propose a scenario that includes several consecutive migrations of sheep populations that may serve as working hypotheses for subsequent studies.

**Electronic supplementary material:**

The online version of this article (doi:10.1186/s12711-015-0139-z) contains supplementary material, which is available to authorized users.

## Background

**Fig. 1:**
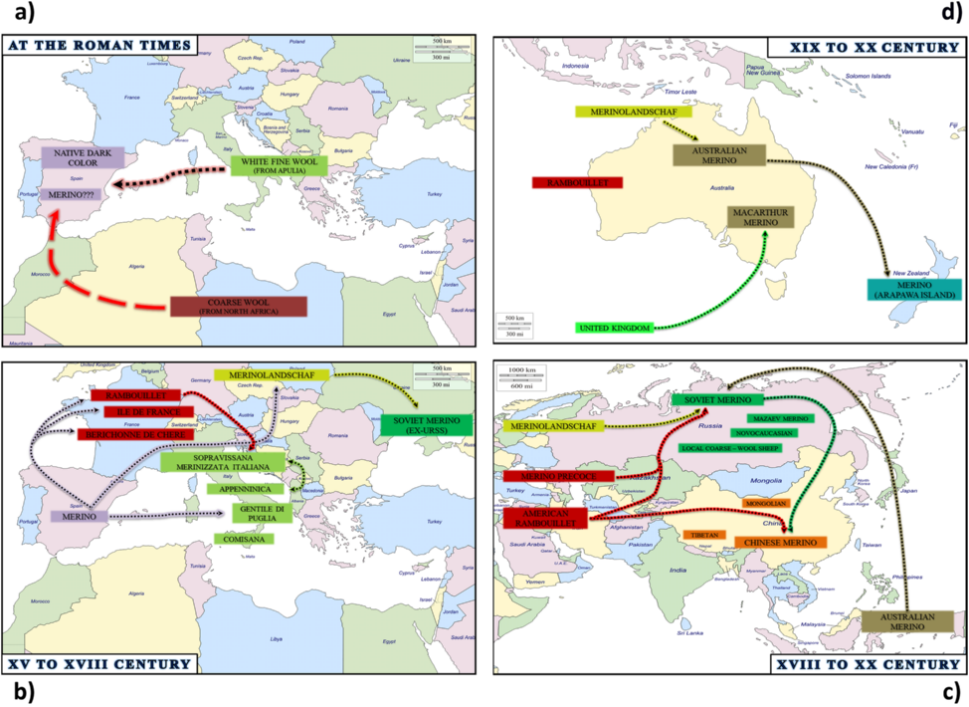
Synoptic scheme that summarizes the hypothetical origin and relationships between Merino and Merino-derived sheep populations at the Roman times (**a**) and from XV to XVIII century (**b**) in the Mediterranean area, from XVIII to XX century in Asia (**c**) and from XIX to XX century in Australia and New Zealand (**d**)

Sheep domestication from its wild ancestor (*Ovis orientalis*) occurred more than 11 000 years B.C. in the Fertile Crescent (south west Asia) [1]. Initially raised for meat production, sheep reached Europe and the Mediterranean regions ca. 6000 B.C. during the Neolithic revolution [2]. Sheep genetic stocks that presented phenotypic traits useful to the fiber (wool) industry were introduced later and most of the modern breeds derived from these [3]. In Europe, the presence of sheep breeds with a primitive appearance that is not adapted to wool production, and of the feral mouflon, is probably a legacy of the early Neolithic sheep wave. During Roman times, Latin authors described different sheep breeds according to their fleece characteristics and celebrated the fine wool of ewes from the Apulia region, in Southern Italy [4]. Columella reported that during the first century B.C. fine-wool ewes from Apulia were introduced in the southern part of Spain (Baetica) and that they were mated with coarse-wool rams from Africa. During the same period, the Greek author Strabo wrote about the beautiful dark color of the Spanish native breeds. These breeds are believed to have been crossed with sheep imported by Arabs and are probably the main ancestor of the Merino breed, which was developed since the late Middle Ages [5] (Fig. 1a).

It is believed that during the 15^th^ century, Merino rams were transferred from Aragon (Spain) to Southern Italy (at that time ruled by an Aragon dynasty), and mated to native ewes, giving rise to the contemporary Gentile di Puglia breed [6]. During the 18^th^ century, purebred Merino rams were exported to Saxony (Germany) and France where they founded the Merinolandschaf and Rambouillet breeds, respectively. The Rambouillet breed was also exported to Central Italy where it gave rise first to the Sopravissana breed [7] and, only more recently through further sporadic introgression, to the Merinizzata Italiana breed. In addition to the above-mentioned Merinolandschaf and Merinizzata Italiana breeds, other breeds were derived from Merino stocks and developed as mutton breeds, such as the French Ile de France and Berrichon du Cher [8] (Fig. 1b). The dispersal of Merino sheep in Eastern Europe also started in the 18^th^ century. In Hungary, Merino sheep breeding began in 1774 when Empress Maria Theresa bought 300 Merino individuals from Spain [9]. In China, several fine-wool breeds were produced by crossing Mongolian and Tibetan ewes with Soviet Merino and Rambouillet rams [10] (Fig. 1c). During the 19^th^ century, the Merino breed was also exported to Australia and New Zealand through successive introductions of sheep from Germany and France. On the Arapawa island (New Zealand), the Merino population has reverted to the feral status (Fig. 1d). During the Soviet era, many composite fine-wool sheep breeds were developed in the vast URSS territory by crossing Australian Merino, American Rambouillet, Merino Précoce and Merinolandschaf with local Merino (Novocaucasian and Mazaev Merino) and/or coarse-wool sheep [11].

In more modern times, Merino and their derived breeds have become cosmopolitan. They played a critical role in the economic development of countries such as Australia through the production of high-quality wool. In contrast, in the last decades, European Merino sheep have experienced a dramatic numerical decrease to the point that they are considered as endangered breeds and no longer undergo active genetic improvement. In this study, we have investigated the genetic structure and relationships among Merino and Merino-derived breeds around the globe, which is relevant for the preservation, management and exploitation of the world-wide Merino genetic variability. A previous study on the relationships among Merino and non-Merino breeds was reported by Kijas et al. [12]. They analyzed 74 breeds from six continents using the Illumina Ovine SNP50 BeadChip (SNP for single nucleotide polymorphism) and detected extensive haplotype sharing between Merino and other breeds, which likely results from the widespread use of Merino sires across Europe in the past centuries. Our study is a follow-up on this, and focuses on the genetic diversity, structure and admixture of Merino and Merino-derived breeds. Towards this aim, we extended the available sheep dataset by adding samples from the original Spanish Merino sheep breed and from additional Merino-derived populations. In addition, we included novel Mediterranean and Northern European breeds which, together with previously unpublished data from feral and wild sheep, contribute to provide new insights into the remote origin of the most famous fine-wool sheep breed in the world.

## Methods

### Genotypic data

We used genotypes that were obtained with the OvineSNP50 BeadChip (Illumina, San Diego, CA) for a total of 37 breeds or populations (671 samples [See Additional file 1: Table S1]), which were selected as representative of the following groups: (1) Merino breeds and breeds known to have undergone significant Merino influence (Spanish Merino, Australian Industry Merino, Australian Merino, Australian Poll Merino, Rambouillet, Merinolandschaf, Chinese Merino, Macarthur Merino, Arapawa, Gentile di Puglia, Sopravissana and Merinizzata Italiana). It should be noted that the animals of the Spanish Merino sample were selected to represent two scenarios of how this breed was derived: a current commercial population (represented by animals sampled in the Estremadura region) and the “historical” Spanish Merino population (represented by animals conserved in the selection centre at Hinojosa del Duque, Cordoba, Andalusia); (2) Spanish breeds with no known Merino influence (Churra, Ojalada, Rasa Aragonesa and Castellana); (3) Italian breeds with no known Merino influence (Massese, Appenninica, Laticauda, Leccese, Comisana and Sardinian White); (4) domestic descendants of primitive sheep (German and Dutch Heath, Scottish Blackface, Finnsheep, Boreray and Soay), as well as feral (Sardinian mouflon and European mouflon) and wild (Asiatic mouflon (*O. orientalis*), argali (*O. ammon*), and urial (*O. vignei*)) sheep populations belonging to the genus *Ovis* [See Additional file 1: Table S1]. For each breed, about 24 animals were sampled from different flocks to avoid closely related individuals. Only three to six samples were available for the six breeds of the German and Dutch Heath sheep (Gray Horned Heath sheep, White Horned Heath sheep, Schoonebeker Heath sheep, Bentheimer sheep, Drenthe Heath sheep, and Veluwe Heath sheep) and for *O. orientalis*, *O. ammon* and *O. vignei*. Genotype data originated from different sources. The BiOvIta project [4] contributed the Italian breeds Gentile di Puglia, Sopravissana, Merinizzata Italiana, Massese, Appenninica, Laticauda, and Sardinian White. Hans Roest and Gijsbert Six (Dutch Drenthe Heath Sheep Breeding Society) [13] provided samples for the six German and Dutch Heath sheep breeds. The Heath sheep and the Spanish Merino were genotyped, as described by Kijas et al. [12], for the specific purposes of this work. Genotypes for the remaining breeds were provided by the International Sheep Genomics Consortium [12]. Raw genotype data were analyzed using the GenomeStudio Genotyping Module v. 1.7 by setting the no-call threshold to 0.15. Moreover, the following filtering parameters were adopted to exclude certain loci and animals and to generate the pruned input file: SNPs with (i) a SNP call rate less than 99 %, (ii) a minor allele frequency (MAF) less than 1 %, and (iii) with a Y-atypical clustering were removed and (iv) animals with more than 10 % missing genotypes were removed. File editing was carried out using PLINK [14]. All genotype data are freely available at the Dryad digital repository (http://dx.doi.org/10.5061/dryad.2p0qf).

### Genetic diversity analysis

Within-breed genetic diversity was estimated with the following parameters that were obtained using PLINK [14]: proportion of polymorphic loci (*P*_*pl*_); genetic diversity (*H*_e_); inbreeding coefficient (*F*). The distribution of the number of SNPs over frequency bins for each population was obtained using PLINK and plotted on a graph using Microsoft Excel. Pair-wise IBS (identical by state) distances among the sheep samples were calculated using PLINK [14] and graphically represented by multi-dimensional scaling (MDS) analysis.

Genetic relationships among breeds and levels of gene flow and admixture were evaluated through the model-based clustering algorithm implemented in the software ADMIXTURE v. 1.22 [15] by applying the default settings. The default (5-fold) ADMIXTURE’s cross-validation procedure was carried out to estimate, for each *K* value (number of assumed clusters), prediction errors. The value of *K* that minimizes this estimated prediction error is assumed to be the most probable. Individual coefficients of membership to each *K* cluster produced by ADMIXTURE were visualized using the program DISTRUCT [16].

Relationships among breeds were also explored by neighbor-network analysis of the (1-IBS) distance [14] and the distances of Nei [17] and Reynolds *et al*. [18] calculated by PowerMarker [19]. Neighbor-networks were constructed using the Neighbor-Net algorithm [20] implemented in the SplitsTree4 package v. 4.13.1 [21].

In order to reconstruct historical relationships between the analyzed populations and to test for the presence of gene flow, we adopted the tree-based approach implemented in TREEMIX [22]. First, the TREEMIX program was run on the dataset described above, with animals classified in 37 breeds or populations. A variable number of migration events (*M*) ranging from 0 to 50 was tested, and the value of *M* that had the highest log-likelihood was selected as the most predictive model. Then, the 37 breeds or populations were grouped into six arbitrary groups (wild, feral, primitive, Merino and Merino-derived, Spanish non-Merino, Italian non-Merino) and the analyses were repeated as previously described. The tests *f3* and *f4* that are implemented in the TREEMIX computer package [22] were also performed on the dataset arranged into the six arbitrary groups. We used the *f3*-statistics (A, B, C) to determine if A was derived from the admixture of populations B and C, and the *f4*-statistics ((A, B,) (C, D)) to test the validity of a hierarchical topology in four-population trees. Significant deviations of the *f4*-statistics from 0 for the three possible topologies of four-population trees are evidence of gene flow in the tree, i.e., that the phylogeny of the four groups is not completely tree-like. A significantly positive *Z*-score indicates gene flow between populations related to either A and C or B and D and a significantly negative *Z*-score indicates gene flow between populations related to A and D or B and C.

To detect pairs of populations that share common ancestry and/or have experienced gene flow, we investigated the extent of pair-wise haplotype sharing with two approaches. The first approach is based on the calculation of correlation coefficients *r* for the same pair of SNPs in two different breeds as measures of linkage disequilibrium (LD) [23]. First, pairs of SNPs were binned in intervals of 0 to 1000, 1000 to 2000 bp and so on. Then, following Kijas *et al*., [12], r coefficients were calculated for pairs of SNPs that were separated by 0 to 10 kb, 10 to 25 kb and 100 to 250 kb, respectively. Breeds for which *r* coefficients increase for SNPs at longer distances are expected to share a more recent common ancestor. To avoid data flattening, out-groups (*O. orientalis, O. argali* and *O. ammon*) were removed from the dataset for this analysis. The second method is based on the direct inference of population haplotypes using fastPHASE [24], a software program that has been shown to perform well even with moderately low LD [25]. Then, for all possible population pairs, haplotype diversity was measured at haplotype blocks that comprised three SNPs. Haplotype blocks for which the inter-population haplotype diversity is less than 0.3 and that are more than 0.5 cM long are considered to be shared by populations. Finally, the length of all shared segments was summed for each pair of populations.

## Results

### Polymorphism and genetic diversity

After data editing, the dataset included 671 samples from 37 breeds or populations that were genotyped for 45 847 SNPs. Within-breed genetic diversity parameters are in Table S1 [See Additional file 1: Table S1]. Distributions of the number of SNPs across frequency bins for all population samples are in Figure S1 [See Additional file 2: Figure S1]. As expected, because selection of SNPs was based on modern domestic breeds, the wild, feral and primitive populations have a lower genetic diversity compared to modern breeds, as suggested by the low values of P_pl_ and *H*_*e*_ [See Additional file 1: Table S1], and by the large proportion of loci that fall in frequency bins between 0.8 and 1 [See Additional file 2: Figure S1]. Measures of genetic diversity for the Merino and Merino-derived breeds were similar to those for other non-Merino European modern breeds [See Additional file 1: Table S1] ([12]). However, the isolated Macarthur Merino population had the lowest genetic diversity (*P*_*pl*_ = 0.617 and *H*_e_ = 0.217). Bias due to sample size was excluded by re-sampling analysis i.e. rarefying each population sample to a maximum of 10 animals (the size of the Macarthur Merino sample) and re-calculating *P*_*p*__*l*_, *H*_*e*_ and *F* [See Additional file 1: Table S2].

### Multi-dimensional scaling analysis

**Fig. 2:**
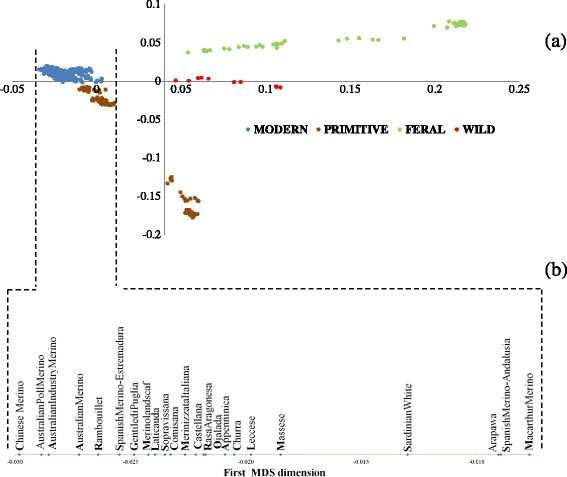
**a** MDS plot of the pair-wise IBS distances among samples that belong to the 37 populations considered. Color codes indicate the four groups to which animals were *a priori* assigned. **b** Position of breeds along the first dimension (*x*-axis) of the MDS plot of the pair-wise IBS distances after zooming on the modern breeds

The MDS plot of the pair-wise IBS distances is in Fig. 2a. A clear gradient along the first dimension was observed, which separated wild and feral sheep (right side) from domestic (left side) sheep. Accordingly, animals that originated from primitive north-European breeds occupied an intermediate position along the *x*-axis. Interestingly, wild and feral individuals were more scattered than their domestic counterparts. Spanish Merino sheep were also more scattered over the plot than other domestic breeds (data not shown).

Regarding the modern breeds (Fig. 2b), they show a cline (from the right to the left side of the plot) from Macarthur Merino, Spanish Merino from Andalusia and Arapawa to Merinolandschaf, Gentile di Puglia, Spanish Merino from Estremadura, Rambouillet, the three Australian Merino populations and Chinese Merino.

### Structure and admixture analysis

**Fig. 3:**
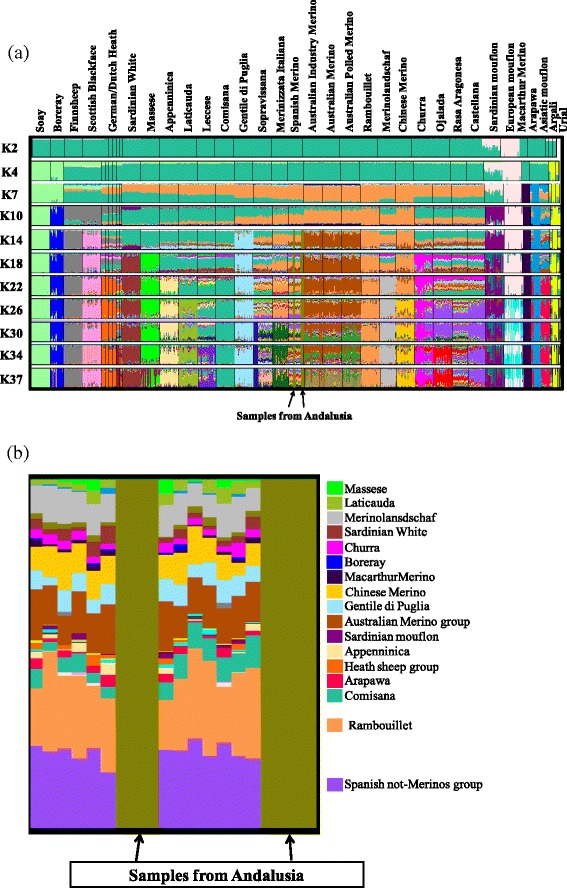
**a** Genetic structure and admixture plot for all breeds analyzed in this study based on coefficients of individual membership to clusters (*K*) assumed to be present in the complete sample. **b** Zoomed detail of the genetic structure and admixture plot for the Spanish Merino breed

Model-based clustering was performed to search for admixture and genetic distinctiveness between breeds and breed groups. The plot obtained by ADMIXTURE analysis is in Fig. 3a, which shows the results for *K* (number of clusters assumed in the whole sample) ranging from 2 to 37. At *K* = 2, the first populations to be differentiated are the Sardinian and the European mouflon, which display a similar pattern of genetic differentiation that confirms the hypothesis of mutual introgression between the Sarda breed and the Sardinian mouflon [4]. At *K* = 4, four distinct genetic components are clearly observed: the three wild sheep (*O. orientalis*, *O. ammon*, and *O. vignei*), the mouflon, the primitive Soay and Boreray breeds, and the modern breeds. At *K* = 10, a Merino genetic component is detected that is shared by the three Australian Merino populations, most of the Merino-derived breeds (Rambouillet, Chinese Merino, Merinolandschaf, and Merinizzata Italiana), Spanish Merino and, partially, by the four Spanish non-Merino breeds (Churra, Ojalada, Rasa Aragonesa, and Castellana) and the Italian Merino-derived breeds Gentile di Puglia and Sopravissana. Another major component is shared by the Italian breeds Massese, Appenninica, Laticauda, Leccese, Comisana, and Sardinian White (also influenced by the Sardinian mouflon), and, partially, by the Gentile di Puglia, Sopravissana, the four Spanish non-Merino breeds, Merinizzata Italiana, Spanish Merino and Merinolandschaf breeds. At *K* values higher than 10, the three Australian Merino populations form a separate cluster and more and more breeds are differentiated.

At the *K* value for which the ADMIXTURE cross-validation error was lowest (*K* = 26; [See Additional file 2: Figure S2]), several breeds appeared to be homogeneous and clearly separated from all others, i.e. Soay, Boreray, Finnsheep, Scottish Blackface, Sardinian White, Massese, Appenninica, Gentile di Puglia, Rambouillet, Merinolandschaf, Chinese Merino and Macarthur Merino. Other breeds showed a simple admixture-like pattern, such as the Laticauda, which is mainly admixed with Leccese and Comisana sheep; the Leccese, which is admixed with Comisana sheep; the Churra, which is admixed with three other Spanish non Merino breeds; and the Sardinian mouflon, which shares a genetic component with Sardinian White and European mouflon. A number of breeds showed a complex admixture-like pattern, notably, Sopravissana, Merinizzata Italiana, Spanish Merino and Rasa Aragonesa. The six German and Dutch Heath sheep belong to the same cluster; the Australian Merino populations were also clustered into a single group; Chinese Merino sheep were linked to the Rambouillet breed, and Arapawa and Spanish Merino displayed a clear sub-structuring. It should be noted that the latter was split into two sub-populations, one that included samples from the Andalusia region and the other samples from the Estremadura region (Fig. 3a and b). In the ADMIXTURE plot, the Merino samples from Andalusia consistently displayed very low *H*_*e*_ values i.e. 0.222 [See Additional file 1: Table S1], which is similar to those observed in our dataset for wild, feral and primitive Northern European sheep (see discussion above), while the *F* value was highly negative (−0.151) [See Additional file 1: Table S1]. In contrast, the Merino sheep from Estremadura had higher *H*_*e*_ values i.e. 0.362) [See Additional file 1: Table S1] and an *F* value close to 0 (−0.033) [See Additional file 1: Table S1].

### Neighbor-network analysis

**Fig. 4:**
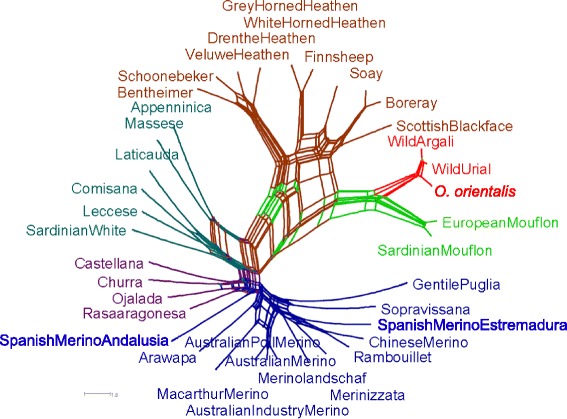
Neighbor-network of Reynolds’ distances

Neighbor-network graphs account for gene flow among breeds (reticulation) and, thus provide a more plausible reconstruction than linear representations. The Neighbor-network clearly separated the wild, feral and primitive North European sheep from modern domestic sheep (Fig. 4). Among these, clear sub-branches were observed that included (i) the Italian Appenninica, Massese, Laticauda, Leccese, Comisana and Sardinian White breeds and (ii) the four Spanish non-Merino breeds (Castellana, Churra, Ojalada and Rasa Aragonesa). The remaining breeds clustered with the group of Merino and Merino-derived breeds. It should be noted that the Spanish Merino from Andalusia occupies a central position between these two sub-branches close to the Spanish non-Merino breeds, while the Estremadura Merino is integrated in the Merino cluster.

### Tree-based migration analysis

Analysis of the TREEMIX log-likelihood values for 0 to 50 migrations revealed that the most predictive model (i.e. that had the highest log-likelihood) assumed the presence of 40 migration events [See Additional file 2: Figure S3]. Table S3 shows which populations are involved in the most consequential migrations and their relative weights [See Additional file 1: Table S3]. In this study, we focused on the migrations that involved Merino or Merino-derived breeds. As expected, strong signals of gene flow and/or shared ancestry were inferred for several Merino and Merino-derived breed pairs, like Merinolandschaf - Rambouillet (0.40); Arapawa with Merinizzata - Macarthur Merino (0.39); Sopravissana - Merinizzata (0.35); Rambouillet - Merinizzata (0.19); the four Australian Merino populations and the New Zealand Arapawa (0.13). A strong signal (0.43) was also observed for the combined two Italian Merino-derived breeds (Gentile di Puglia and Sopravissana) and the combined Italian non-Merino breeds (Massese, Appenninica and Leccese). Similarly, a rather strong signal indicated a gene flow between the German Merinolandschaf and the primitive northern European breeds. This suggests that a few Merino-derived breeds, like Gentile di Puglia, Sopravissana and Merinolandschaf, have conserved traces of the original breed before incrossing with Merino. Besides being influenced by Australian Merino, the Arapawa breed generated a strong signal of gene flow (0.34) from the three Scottish breeds, which suggests that this breed has a composite origin. Interestingly, two migration events that involved large aggregates of breeds and correspond to basal positions in the TREEMIX inferred tree were detected with migrations weights of 0.22 and 0.18, respectively [See Additional file 1: Table S3], which suggests that gene flow also occurred among archaic domestic sheep stocks. The TREEMIX analysis was repeated on a dataset in which populations were arranged into six groups corresponding to the most likely archaic sheep stocks (wild, feral, primitive, Merino, Spanish non-Merino, Italian non-Merino) and for which Merino-derived breeds (Chinese Merino, Merinolandschaf, Sopravissana, Gentile di Puglia and Merinizzata) were removed in order to avoid confounding of the analysis by more recent events. Analysis of the TREEMIX log-likelihood values for the aggregated dataset and for each tested scenario (*M* = 0, 1, 2, 3, 4, 5, 10, 20, 30, 40, 50) revealed that the most predictive model assumed four migration events [see Additional file 2: Figure S4]. The highest signals of migration and gene flow [See Additional file 1: Table S4] were observed between Merino and feral sheep (0.19), and between Merino and primitive sheep (0.18). In order to verify whether the above scenario may have been influenced by the presence, in the Merino group, of Spanish Merino samples from Andalusia (which, in the above MDS analysis, were shown to be close to primitive, feral and wild sheep), as well as by the recently admixed Arapawa and the inbred Macarthur Merino breeds, we removed these samples. However, the signal indicating a gene flow between the Merino group and the wild/feral group remained [See Additional file 1: Table S5].

The *f3* test on the aggregated dataset did not produce any significant *Z*-score (data not shown). The *f4* test highlighted highly significant *Z*-scores (>3) for three four-population trees [See Additional file 1: Table S6]. Admixture signals were observed between Merino and Spanish non-Merino breeds, Spanish non-Merino and Italian breeds, Italian and wild, Italian and primitive, wild and feral, Merino and wild, Merino and primitive sheep.

### Pair-wise haplotype sharing

Heat maps of pair-wise haplotype sharing, which were estimated either by correlating *r* values across breeds or by direct count of shared blocks reconstructed *in silico*, are in Figures S5, S6, S7 and S8 [See Additional file 2: Figures S5, S6, S7 and S8]. The results from both methods were in good agreement and generally consistent with the results described above. As expected because of their history, haplotype sharing by the three Australian Merino populations is always by far the highest. The across-breed correlation for pairs of SNPs that were separated by 0 to 10 kb and 10 to 25 kb produced almost identical patterns [See Additional file 2: Figures S5 and S6]. Haplotype sharing with other breeds was low for sheep from the Heath group. Most of the remaining primitive and feral populations had a moderate mutual haplotype sharing, but it was clearly higher for the other breeds (Merino and Merino-derived; Spanish non-Merino; Italian non-Merino). A different pattern was observed for pairs of SNPs that were separated by 100 to 250 kb [See Additional file 2: Figure S7], with haplotype sharing found only for Merino and Merino-derived breeds. A pattern with two groups was also observed if haplotype sharing was based on direct counts of shared blocks [See Additional file 2: Figure S8]. This pattern also confirmed (i) the “primitive” nature of the Andalusian Spanish Merino and Macarthur Merino breeds, as previously suggested by the MDS analysis, and (ii) the Scottish Blackface influence on the Arapawa breed, as previously observed using the TREEMIX approach. However, it is interesting to note that the Arapawa breed clustered with the three Australian Merino breeds for pairs of SNPs that were separated by 100 to 250 kb [See Additional file 2: Figure S7].

## Discussion

### Genetic diversity

Analysis of a comprehensive dataset of wild, feral, primitive and Merino and non-Merino breeds allowed us to gain insight in the history of the world-wide sheep population. Although our data revealed inbreeding in the Macarthur and Andalusian Merino populations, the low *H*_*e*_ values found for wild, feral and primitive sheep reflect the fact that these breeds were not used to identify polymorphic SNPs for the OvineSNP50 BeadChip (Illumina) (SNP ascertainment bias [12]) rather than a low genomic diversity. Interestingly, high levels of variability were still present in the Spanish Merino population from Estremadura, in spite of a marked decrease in census size in the last 50 years (−18.9 % between 1955 and 1974 and −36.8 % between 1970 and 1986 [26]). This variability may have been influenced by cross-breeding with Merino-derived breeds since the 1960s to direct the Merino breed towards meat production.

### Relationships among Merino sheep and other breed groups

Our data indicate a clear and consistent subdivision of the breeds considered in this study. The MDS plot highlighted four groups corresponding to modern, primitive, feral and wild sheep. This agreed with the four main branches in the neighbor-network and the model-based clustering at *K* = 4 groups. Overall, haplotype sharing showed more cross-flow and relatedness between Merino and Merino-derived breeds than between other sheep populations. Our evidence points to admixture events during different time periods. A breed-based TREEMIX analysis detected two basal migrations that involved large assemblages of breeds, which is consistent with the gene flow that occurred between archaic domestic sheep stocks. Signals of gene-flow among feral, primitive and Merino sheep populations were also observed when the TREEMIX analysis was performed on the dataset arranged into the six arbitrary groups. In addition, the four-population test produced strong evidence to support migration between Spanish populations and both Merino and Italian populations. Since this cannot be readily explained by recent gene flow, these signals may indicate remote admixture, which may be the combined effect of documented export of wool sheep from Roman Italy to Spain and late-Medieval export of sheep from Spain to Southern Italy under the Aragonese rule [6]. As discussed below, Spanish Merino sheep were exported since the Middle Ages to many countries and crossed with local sheep. Since the genetic distances estimated in our study demonstrate a closer relationship between all Spanish modern breeds than between Spanish breeds and other Mediterranean sheep, this suggests that the selection of Merino sheep to produce fine wool did not result in a genetic differentiation from other Spanish sheep that were bred for different purposes.

### Origin of the Merino breed

For many years, several authors have addressed the question of when and how the Merino breed developed [27]. Some hypotheses invoke the role of different importation events of sheep stocks into Spain to improve local breeds (i) during the Roman era from Southern Italy (Apulian sheep, [28]) which can still be traced in the current Gentile di Puglia sheep; (ii) during the Moorish colonization from Northern Africa (Berber sheep); and (iii) during the 15^th^ century from England (fine wool sheep, most likely the Cotswold breed).

To obtain an unambiguous picture of the ancient genetic relationships between the breeds in our dataset and, thus gain insight into the origin of the Merino breed is not straightforward. A clear Merino genetic component was detected in several breeds at *K* = 10 in the ADMIXTURE analysis, which provides evidence for an extensive circulation of Merino genetic material as previously reported by Kijas *et al.* [12], but confounds a reconstruction of ancestral breed relationships. However, the close relationship between the Merino breed and other Spanish modern breeds as shown in the neighbor-network graph and the admixture between the Merino breed and other Spanish breeds as revealed by the four-population test, both support an Iberian origin. This is in agreement with the documented late-Medieval origin of the Merino breed, but is also compatible with a partial Italian ancestry that would correspond to the documented gene flow during the Roman period.

### On the modern Merino and the merinization

In our study, a marked differentiation between the Spanish Merino populations from Estremadura and from Andalusia was observed with both the ADMIXTURE and the neighbor-network analyses. This likely reflects the isolation of animals reared at the governmental selection center of Hinojosa del Duque (Cordoba, Andalusia), where, in 1971, animals from the five main traditional Spanish Merino genetic lines were collected to ensure the conservation of purebred animals [29]. The ADMIXTURE component observed in the Andalusian Merino was not shared by any of the other Merino or Merino-derived breeds (including the Spanish Merino samples from Estremadura) and may be considered as a typical inbreeding signal, which was also observed for other inbred breeds (Fig. 2a) [30]. The fact that mating among relatives within color types (line breeding) was avoided [29] probably explains the negative *F* values observed in our sample [31]. It is interesting to note that the specific genetic make-up of the sheep from Hinojosa del Duque revealed by the ADMIXTURE analysis was associated to the fact that these animals were very close to the large cluster that included wild, feral and Northern European primitive sheep as shown in the MDS plot and in the network analysis using the (1-IBS) distance (data not shown), as well as in the various scenarios tested in the haplotype sharing analysis. This result supports the hypothesis that these sheep from Hinojosa del Duque may represent remnants of a more archaic Merino stock.

In agreement with the known history of the breeds and with previous studies [12], the ADMIXTURE analysis detected, at *K* = 7 and *K* = 10, clear signals of merinization in European and Australian breeds. This was also clearly confirmed by the TREEMIX analysis. A strong merinization signal was detected by ADMIXTURE analysis in the Rambouillet breed, which was developed in France directly from pure Spanish Merino imported in the Rambouillet region during the second half of the 18^th^ century. Remarkably, a less intense Merino signal was detected in the Australian Merino. Although different Merino stocks from Cape Colony, England, Saxony, France and America are known to have contributed to the early development of this breed and although different Merino strains are currently bred in Australia, the three Australian Merino populations that were tested here have homogeneous patterns in the ADMIXTURE analysis, low pair-wise IBS and Reynolds distances and share many haplotypes. This reflects a common distinctiveness from all the other Merino populations considered in this study, which originates from a shared history after their arrival in Australia. The ADMIXTURE patterns at various *K* values confirm that the Rambouillet breed contributed to the development of Chinese Merino. Breeds, such as Sopravissana, Merinizzata Italiana, Merinolandschaf and Gentile di Puglia, which are known or believed to have been cross-bred to Merino sires still have traces of their autochthonous origin, as suggested by ADMIXTURE and TREEMIX analyses. Macarthur Merino has a low *H*_*e*_ (see also [32]) and separates from all the other breeds, including the three other Australian Merino populations at low *K* values. This is consistent with its history of inbreeding within a closed nucleus that was developed since the early 19^th^ century mainly from English Merino sheep. Thus, the world-wide Merino population has been formed by intensive gene flow, local ancestry, founder effects and geographic isolation.

ADMIXTURE patterns confirm the clear subdivision of the Arapawa island breed [33]. In agreement with [33], our results support the hypothesis that this breed is the result of contributions from multiple stocks. The neighbor-network topology and the haplotype sharing detected by pairs of SNPs that are separated by 100 to 250 kb indicate that the Australian Merino had a major role in the genetic makeup of this breed. TREEMIX analysis also suggested a Scottish influence, which is consistent with the fact that Scottish Blackface sheep were imported into New Zealand at the end of the 19^th^ century [34].

## Conclusions

This study represents an in-depth genome-wide analysis of Merino and Merino-derived breeds. It provides a general picture of the relationships between Merino and Merino-derived breeds on the one hand, and wild, feral, primitive and other modern domestic sheep, on the other. It contributes to the reconstruction of the history of merinization, which involved many sheep breeds world-wide during the last three centuries. We showed that sub-structuring has affected the current Spanish Merino genetic makeup, which may be relevant for breed management and conservation. Our results support the hypothesis that the Spanish Merino breed has an Iberian origin without excluding ancient contributions from other Mediterranean stocks. Together our results fit with the proposed scenario that describes how this breed may have originated, which is also partially supported by ancient and more recent documentation: (i) admixture among the early domesticated sheep, which explains the shared ancestry of modern Mediterranean breeds; (ii) export of Italian wool sheep during the Roman era into Iberia and other parts or the Empire, later possibly followed by African imports into Iberia; and (iii) a relatively recent circulation of Merino material around the world, which contributed to shape a new, genetically distinct, and world-wide disseminated sheep group that combines both Merino and local ancestry. Each of the steps of this scenario can provide working hypotheses for future studies on modern as well as ancient sheep DNA using advanced genomic methodologies.

## Additional files

Additional file 1: 
**Table S1.** Breed and population details and within-population genetic diversity parameters. Table S1 provides, for each considered population, information concerning the species, the geographical origin, the membership of breeds to four groups, as specified in the Methods section, the number of individuals (N), the proportion of polymorphic loci (*P*_*p**l*_), a measure of gene diversity (*H*_*e*_), and the inbreeding coefficient (*F*). **Table S2.** Within-population genetic diversity parameters estimated for rarefied samples. Table S2 provides the proportion of polymorphic loci (*P*_*p**l*_), a measure of gene diversity (*H*_*e*_), and the inbreeding coefficient (*F*) for population samples rarefied to *N* = 10 each. The membership of breeds to four groups, as specified in the Methods section, is also indicated. **Table S3.** Breeds and populations involved in the migration events inferred by TREEMIX. Table S3 provides a list of breeds and populations for which TREEMIX detected possible migration events. The analysis was carried out using the dataset of 671 samples from 37 populations, assuming *M* = 40. Relative migration weights are indicated. Branches of trees are shown in the Newick format. Only migrations with weights greater than 0.1 are shown. **Table S4.** Groups of populations involved in the migration events inferred by TREEMIX. Table S4 shows the results of the TREEMIX analysis carried out after removal of the Merino-derived breeds (Chinese Merino, Merinolandschaf, Sopravissana, Gentile di Puglia and Merinizzata) and clustering of the considered breeds and populations into six arbitrary groups, as specified in the Methods section. Relative migration weights are indicated. **Table S5.** Groups of populations involved in the migration events inferred by TREEMIX, after removal of the Andalusian Merino, Arapawa and Macarthur Merino populations. Table S5 shows the results of the TREEMIX analysis carried out after removal of Andalusian Merino, Arapawa, Macarthur Merino and the Merino-derived breeds (Chinese Merino, Merinolandschaf, Sopravissana, Gentile di Puglia and Merinizzata) and clustering of the considered breeds and populations into six arbitrary groups, as specified in the Methods section. Relative migration weights are indicated. **Table S6.** *Z*-scores from the *f4* test on the aggregated dataset. Table S6 shows the results of the *f4* test carried out using the dataset aggregated into six arbitrary groups (wild, WIL; feral, FER; primitive, PRIM; Merino, MER; Spanish non-Merino, SPA; Italian non-Merino; ITA). Only trees with significant *Z*-scores (>3) are shown. For each tree, the three possible four-population topologies are reported. Additional file 2: 
**Figure S1.** Distributions of the number of SNPs across frequency bins for all population samples. Breeds are ordered along the *x*-axis according to group membership (Merino and Merino-derived sheep, in blue; Spanish non-Merino sheep, in purple; Italian non-Merino sheep, in cyan blue; primitive North European sheep, in brown; feral sheep, in green; wild sheep, in red). **Figure S2.** ADMIXTURE cross-validation analysis. For each number of assumed clusters (*K*) ranging from 1 to 37, prediction errors were calculated from five independent runs. **Figure S3.** TREEMIX log-likelihood values for the dataset of 671 samples arranged in 37 populations and for different numbers of migrations. **Figure S4.** TREEMIX log-likelihood values for the aggregated dataset with populations arranged into six groups as specified in the Methods section, and for different numbers of migrations. **Figure S5.** Heat map showing the correlation of *r* for pairs of SNPs that are separated by 0 to10 kb. **Figure S6.** Heat map showing the correlation of *r* for pairs of SNPs that are separated by 10 to 25 kb. **Figure S7.** Heat map showing the correlation of *r* for pairs of SNPs that are separated by 100 to 250 kb distances. **Figure S8.** Heat map showing the pair-wise haplotype sharing distances, calculated as the logarithm of 1/(total length of shared segments across the genome).
