# Supplementary material for: Merino and Merino-derived sheep breeds: a genome-wide intercontinental study
Source: Genet Sel Evol. 2015 Aug 14;47(1):64. doi: 10.1186/s12711-015-0139-z (PMC4536749; doi:10.1186/s12711-015-0139-z)
Supplement: Additional file 1: — Table S1. Breed and population details and within-population genetic diversity parameters. Table S1 provides, for each considered population, information concerning the species, the geographical origin, the membership of breeds to four groups, as specified in the Methods section, the number of individuals (N), the proportion of polymorphic loci (P pl), a measure of gene diversity (H e), and the inbreeding coefficient (F). Table S2. Within-population genetic diversity parameters estimated for rarefied samples. Table S2 provides the proportion of polymorphic loci (P pl), a measure of gene diversity (H e), and the inbreeding coefficient (F) for population samples rarefied to N = 10 each. The membership of breeds to four groups, as specified in the Methods section, is also indicated. Table S3. Breeds and populations involved in the migration events inferred by TREEMIX. Table S3 provides a list of breeds and populations for which TREEMIX detected possible migration events. The analysis was carried out using the dataset of 671 samples from 37 populations, assuming M = 40. Relative migration weights are indicated. Branches of trees are shown in the Newick format. Only migrations with weights greater than 0.1 are shown. Table S4. Groups of populations involved in the migration events inferred by TREEMIX. Table S4 shows the results of the TREEMIX analysis carried out after removal of the Merino-derived breeds (Chinese Merino, Merinolandschaf, Sopravissana, Gentile di Puglia and Merinizzata) and clustering of the considered breeds and populations into six arbitrary groups, as specified in the Methods section. Relative migration weights are indicated. Table S5. Groups of populations involved in the migration events inferred by TREEMIX, after removal of the Andalusian Merino, Arapawa and Macarthur Merino populations. Table S5 shows the results of the TREEMIX analysis carried out after removal of Andalusian Merino, Arapawa, Macarthur Merino and the Merino-derived breeds (Chinese [file 12711_2015_139_MOESM1_ESM.zip › Additional file 1/Table S2.pdf]

**Breed name (N = 10)**	**Group**	P_{pl}	H_e	**F**
Spanish Merino (Estremadura)	1	0.955	0.359	0.047
Spanish Merino (Andalusia)	1	0.642	0.222	0.273
Australian Merino	1	0.949	0.357	0.075
Australian Poll Merino	1	0.939	0.349	0.054
Australian Industry Merino	1	0.949	0.356	0.053
Rambouillet	1	0.932	0.341	0.127
Merinolandschaf	1	0.927	0.341	0.078
Chinese Merino	1	0.935	0.344	0.064
Macarthur Merino	1	0.621	0.219	0.406
Arapawa	1	0.853	0.292	0.258
Gentile di Puglia	1	0.934	0.345	0.053
Sopravissana	1	0.940	0.348	0.088
Merinizzata	1	0.952	0.356	0.061
Churra	2	0.931	0.341	0.086
Ojalada	2	0.956	0.360	0.029
Castellana	2	0.957	0.362	0.036
Rasa Aaragonesa	2	0.966	0.367	0.013
Massese	3	0.923	0.337	0.091
Appenninica	3	0.940	0.349	0.054
Laticauda	3	0.941	0.350	0.095
Leccese	3	0.946	0.353	0.086
Comisana	3	0.944	0.352	0.299
Sardinian White	3	0.928	0.342	0.099
Scottish Blackface	4	0.915	0.334	0.076
Finnsheep	4	0.917	0.337	0.136
Soay	4	0.745	0.253	0.325
Boreray	4	0.775	0.261	0.324
Schoonebeker Heath sheep	4	0.741	0.281	0.183
Bentheimer sheep	4	0.816	0.304	0.137
Drenthe Heath sheep	4	0.821	0.306	0.123
Veluwe Heath sheep	4	0.826	0.310	0.109
European mouflon	4	0.621	0.207	0.522
Sardinian mouflon	4	0.877	0.304	0.152
Asiatic mouflon	4	0.648	0.254	0.372
argali	4	0.358	0.102	0.734
urial	4	0.511	0.197	0.664
