# Supplementary material for: Merino and Merino-derived sheep breeds: a genome-wide intercontinental study
Source: Genet Sel Evol. 2015 Aug 14;47(1):64. doi: 10.1186/s12711-015-0139-z (PMC4536749; doi:10.1186/s12711-015-0139-z)
Supplement: Additional file 1: — Table S1. Breed and population details and within-population genetic diversity parameters. Table S1 provides, for each considered population, information concerning the species, the geographical origin, the membership of breeds to four groups, as specified in the Methods section, the number of individuals (N), the proportion of polymorphic loci (P pl), a measure of gene diversity (H e), and the inbreeding coefficient (F). Table S2. Within-population genetic diversity parameters estimated for rarefied samples. Table S2 provides the proportion of polymorphic loci (P pl), a measure of gene diversity (H e), and the inbreeding coefficient (F) for population samples rarefied to N = 10 each. The membership of breeds to four groups, as specified in the Methods section, is also indicated. Table S3. Breeds and populations involved in the migration events inferred by TREEMIX. Table S3 provides a list of breeds and populations for which TREEMIX detected possible migration events. The analysis was carried out using the dataset of 671 samples from 37 populations, assuming M = 40. Relative migration weights are indicated. Branches of trees are shown in the Newick format. Only migrations with weights greater than 0.1 are shown. Table S4. Groups of populations involved in the migration events inferred by TREEMIX. Table S4 shows the results of the TREEMIX analysis carried out after removal of the Merino-derived breeds (Chinese Merino, Merinolandschaf, Sopravissana, Gentile di Puglia and Merinizzata) and clustering of the considered breeds and populations into six arbitrary groups, as specified in the Methods section. Relative migration weights are indicated. Table S5. Groups of populations involved in the migration events inferred by TREEMIX, after removal of the Andalusian Merino, Arapawa and Macarthur Merino populations. Table S5 shows the results of the TREEMIX analysis carried out after removal of Andalusian Merino, Arapawa, Macarthur Merino and the Merino-derived breeds (Chinese [file 12711_2015_139_MOESM1_ESM.zip › Additional file 1/Table_S1.pdf]

Species	Breed name	Breed origin	Group	N	P_{pl}	H_e	F
O. aries	Spanish Merino	Spain	1	20	0,973	0,356	0,067
O. aries	Spanish Merino (Estremadura)	Spain	1	13	0,965	0,362	-0,033
O. aries	Spanish Merino (Andalusia)	Spain	1	7	0,676	0,222	-0,151
O. aries	Australian Merino	Australia	1	24	0,982	0,369	0,027
O. aries	Australian Poll Merino	Australia	1	24	0,980	0,365	-0,015
O. aries	Australian Industry Merino	Australia	1	24	0,981	0,368	-0,007
O. aries	Rambouillet	France	1	24	0,974	0,354	0,014
O. aries	Merinolandschaf	Germany	1	21	0,967	0,354	-0,024
O. aries	Chinese Merino	China	1	23	0,972	0,354	-0,039
O. aries	MacArthur Merino	Australia	1	10	0,617	0,217	-0,064
O. aries	Arapawa	New Zealand	1	24	0,957	0,338	0,060
O. aries	Gentile di Puglia	Italy	1	24	0,964	0,351	-0,036
O. aries	Sopravissana	Italy	1	24	0,984	0,371	0,009
O. aries	Merinizzata Italiana	Italy	1	20	0,980	0,366	-0,012
O. aries	Churra	Spain	2	24	0,977	0,359	-0,015
O. aries	Ojalada	Spain	2	24	0,985	0,370	-0,020
O. aries	Castellana	Spain	2	22	0,979	0,379	-0,029
O. aries	Rasa Aragonesa	Spain	2	20	0,988	0,377	-0,022
O. aries	Massese	Italy	3	24	0,961	0,350	-0,018
O. aries	Appenninica	Italy	3	24	0,976	0,359	-0,030
O. aries	Laticauda	Italy	3	24	0,980	0,365	0,002
O. aries	Leccese	Italy	3	23	0,980	0,366	0,041
O. aries	Comisana	Italy	3	24	0,977	0,362	-0,030
O. aries	Sardinian White	Italy	3	24	0,963	0,351	-0,023
O. aries	Scottish Blackface	United Kingdom	4	24	0,963	0,347	-0,051
O. aries	Finnsheep	Finland	4	24	0,960	0,350	0,014
O. aries	Soay	United Kingdom	4	24	0,789	0,259	-0,029
O. aries	Boreray	United Kingdom	4	17	0,805	0,262	-0,021
O. aries	Gray Horned Heath sheep	Germany	4	4	0,727	0,274	-0,154
O. aries	White Horned Heath sheep	Germany	4	3	0,738	0,229	-0,099
O. aries	Schoonebeker Heath sheep	The Netherland	4	4	0,738	0,281	-0,140
O. aries	Bentheimer sheep	Germany	4	5	0,812	0,303	-0,112
O. aries	Drenthe Heath sheep	The Netherland	4	5	0,818	0,306	-0,123
O. aries	Veluwe Heath sheep	The Netherland	4	5	0,823	0,309	-0,127
O. aries musimon	European mouflon	Corsica (France)	4	24	0,698	0,213	0,089
O. aries musimon	Sardinian mouflon	Sardinia (Italy)	4	24	0,916	0,310	-0,078
O. orientalis	Asiatic mouflon	Unknown	4	3	0,642	0,253	0,039
O. ammon	argali	Unknown	4	6	0,355	0,101	-0,026
O. vignei	urial	Unknown	4	4	0,508	0,196	0,330
