# Supplementary material for: Merino and Merino-derived sheep breeds: a genome-wide intercontinental study
Source: Genet Sel Evol. 2015 Aug 14;47(1):64. doi: 10.1186/s12711-015-0139-z (PMC4536749; doi:10.1186/s12711-015-0139-z)
Supplement: Additional file 1: — Table S1. Breed and population details and within-population genetic diversity parameters. Table S1 provides, for each considered population, information concerning the species, the geographical origin, the membership of breeds to four groups, as specified in the Methods section, the number of individuals (N), the proportion of polymorphic loci (P pl), a measure of gene diversity (H e), and the inbreeding coefficient (F). Table S2. Within-population genetic diversity parameters estimated for rarefied samples. Table S2 provides the proportion of polymorphic loci (P pl), a measure of gene diversity (H e), and the inbreeding coefficient (F) for population samples rarefied to N = 10 each. The membership of breeds to four groups, as specified in the Methods section, is also indicated. Table S3. Breeds and populations involved in the migration events inferred by TREEMIX. Table S3 provides a list of breeds and populations for which TREEMIX detected possible migration events. The analysis was carried out using the dataset of 671 samples from 37 populations, assuming M = 40. Relative migration weights are indicated. Branches of trees are shown in the Newick format. Only migrations with weights greater than 0.1 are shown. Table S4. Groups of populations involved in the migration events inferred by TREEMIX. Table S4 shows the results of the TREEMIX analysis carried out after removal of the Merino-derived breeds (Chinese Merino, Merinolandschaf, Sopravissana, Gentile di Puglia and Merinizzata) and clustering of the considered breeds and populations into six arbitrary groups, as specified in the Methods section. Relative migration weights are indicated. Table S5. Groups of populations involved in the migration events inferred by TREEMIX, after removal of the Andalusian Merino, Arapawa and Macarthur Merino populations. Table S5 shows the results of the TREEMIX analysis carried out after removal of Andalusian Merino, Arapawa, Macarthur Merino and the Merino-derived breeds (Chinese [file 12711_2015_139_MOESM1_ESM.zip › Additional file 1/Table_S5.pdf]

Weights	Group A	Group B
0.155	ITALIAN	WILD
0.138	(WILD, FERAL)	MERINO
0.006	FERAL	SPANISH
