# Supplementary material for: Merino and Merino-derived sheep breeds: a genome-wide intercontinental study
Source: Genet Sel Evol. 2015 Aug 14;47(1):64. doi: 10.1186/s12711-015-0139-z (PMC4536749; doi:10.1186/s12711-015-0139-z)
Supplement: Additional file 2: — Figure S1. Distributions of the number of SNPs across frequency bins for all population samples. Breeds are ordered along the x-axis according to group membership (Merino and Merino-derived sheep, in blue; Spanish non-Merino sheep, in purple; Italian non-Merino sheep, in cyan blue; primitive North European sheep, in brown; feral sheep, in green; wild sheep, in red). Figure S2. ADMIXTURE cross-validation analysis. For each number of assumed clusters (K) ranging from 1 to 37, prediction errors were calculated from five independent runs. Figure S3. TREEMIX log-likelihood values for the dataset of 671 samples arranged in 37 populations and for different numbers of migrations. Figure S4. TREEMIX log-likelihood values for the aggregated dataset with populations arranged into six groups as specified in the Methods section, and for different numbers of migrations. Figure S5. Heat map showing the correlation of r for pairs of SNPs that are separated by 0 to10 kb. Figure S6. Heat map showing the correlation of r for pairs of SNPs that are separated by 10 to 25 kb. Figure S7. Heat map showing the correlation of r for pairs of SNPs that are separated by 100 to 250 kb distances. Figure S8. Heat map showing the pair-wise haplotype sharing distances, calculated as the logarithm of 1/(total length of shared segments across the genome). [file 12711_2015_139_MOESM2_ESM.zip › Additional file 2/Figure_S7.pdf]

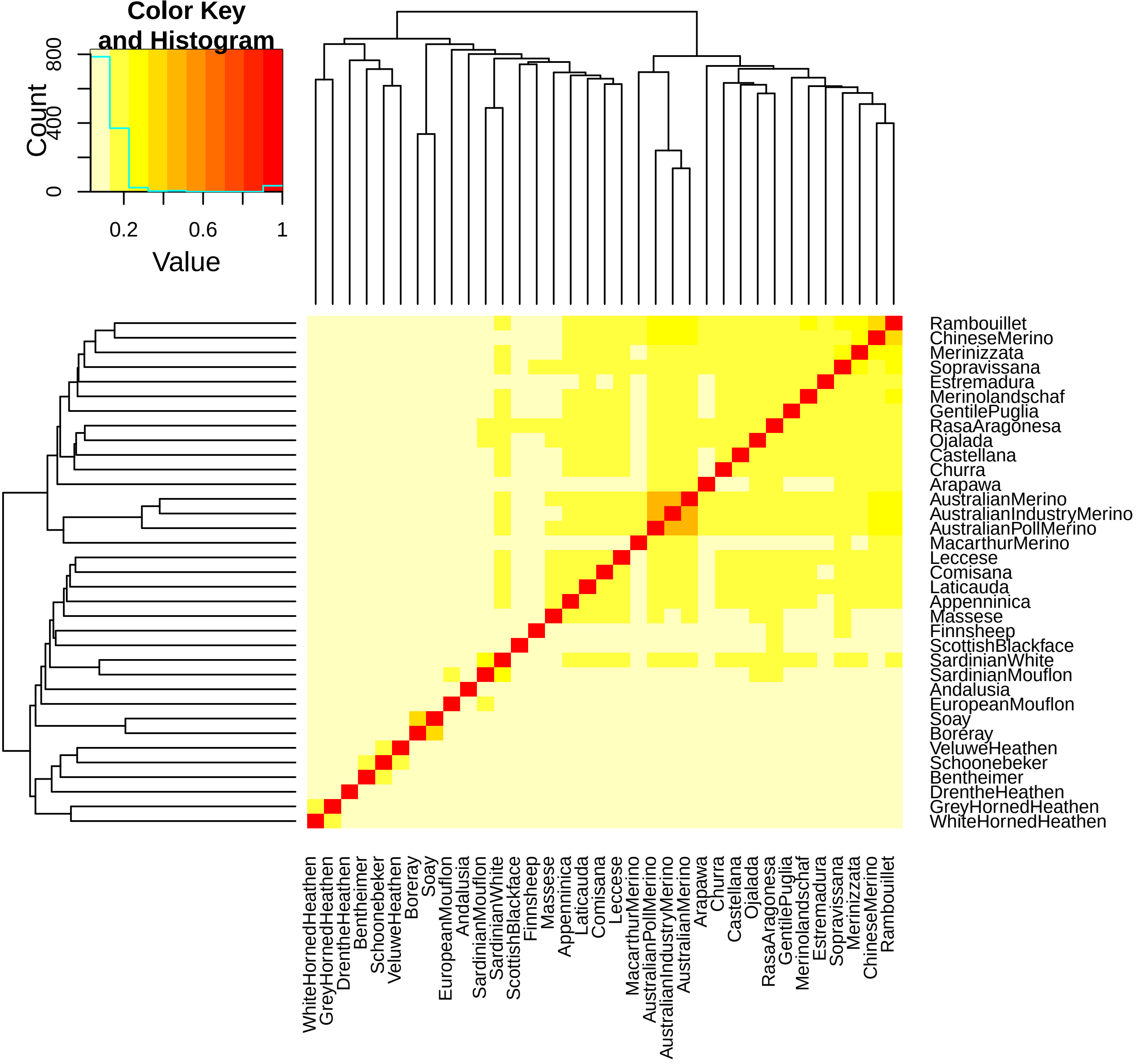

Color Key
and Histogram
Count
0
400
800
0.2
0.6
1
Value
Rambouillet
ChineseMerino
Merinizzata
Sopravissana
Estremadura
Merinolandschaf
GentilePuglia
RasaAragonesa
Ojalada
Castellana
Churra
Arapawa
AustralianMerino
AustralianIndustryMerino
AustralianPollMerino
MacarthurMerino
Leccese
Comisana
Laticauda
Appenninica
Massese
Finnsheep
ScottishBlackface
SardinianWhite
SardinianMouflon
Andalusia
EuropeanMouflon
Soay
Boreray
VeluweHeathen
Schoonebeker
Bentheimer
DrentheHeathen
GreyHornedHeathen
WhiteHornedHeathen
